# Advantageous developmental outcomes of advancing paternal age

**DOI:** 10.1038/tp.2017.125

**Published:** 2017-06-20

**Authors:** M Janecka, F Rijsdijk, D Rai, A Modabbernia, A Reichenberg

**Affiliations:** 1 Social, Genetic and Developmental Psychiatry Centre, Institute of Psychiatry, Psychology and Neuroscience, King’s College London, London, UK; 2 Seaver Autism Center for Research and Treatment, Department of Psychiatry, Icahn School of Medicine at Mount Sinai, New York, NY, USA; 3 Centre for Academic Mental Health, School of Social and Community Medicine, University of Bristol, Bristol, UK; 4 Department of Preventive Medicine, Icahn School of Medicine at Mount Sinai, New York, NY, USA

## Abstract

Advanced paternal age (APA) at conception has been associated with negative outcomes in offspring, raising concerns about increasing age at fatherhood. Evidence from evolutionary and psychological research, however, suggests possible link between APA and a phenotypic advantage. We defined such advantage as educational success, which is positively associated with future socioeconomic status. We hypothesised that high IQ, strong focus on the subject of interest and little concern about ‘fitting in’ will be associated with such success. Although these traits are continuously distributed in the population, they cluster together in so-called ‘geeks’. We used these measures to compute a ‘geek index’ (GI), and showed it to be strongly predictive of future academic attainment, beyond the independent contribution of the individual traits. GI was associated with paternal age in male offspring only, and mediated the positive effects of APA on education outcomes, in a similar sexually dimorphic manner. The association between paternal age and GI was partly mediated by genetic factors not correlated with age at fatherhood, suggesting contribution of *de novo* factors to the ‘geeky’ phenotype. Our study sheds new light on the multifaceted nature of the APA effects and explores the intricate links between APA, autism and talent.

## Introduction

Advanced paternal age (APA) at conception has been associated with a number of adverse neuropsychiatric outcomes in offspring, particularly autism spectrum disorders^1, 2^ and schizophrenia.^3, 4^ Although well replicated, it is plausible that these observations reflect only one side of the nature of the APA effects. Given that historically, conceiving a child later in life was indicative of biological fitness,^5^ there could also exist a link between APA and a phenotypic advantage. In an environment characterised by high mortality, only the fittest males would survive and be able to reproduce when old,^6^ passing their advantageous traits onto offspring. Alternatively, more recent indices of ‘fitness’—including extended educational and career pursuits—could themselves correlate with an older age at fatherhood. Either of these selection mechanisms could help offset negative effects of *de novo* genetic mutations that accumulate in paternal sperm with age.^7^

Despite broad evidence concerning negative consequences of early parenthood, the potential advantage of APA has received very little attention. Very young parents tend to be characterised by lower education and economic disadvantage,^8^ and their offspring were shown to be at increased risk for educational underachievement, crime, substance abuse and health problems.^9^ Although the effects of early reproduction are likely qualitatively different from those of APA,^10^ it may seem surprising that the possibility of protective effects of delayed parenthood has thus far received so little attention.

Here we investigated whether having an older father is associated with certain beneficial traits. We defined such advantageous phenotype based on considerations of what could be ‘adaptive’ in the modern environment. We hypothesised that high IQ; ability to retain strong focus on the subject of interest; and some degree of social aloofness are likely to be particularly beneficial in the knowledge-driven economy. Although these traits are continuously distributed in the population, ethnographic literature groups them under an umbrella-term ‘geek’ (‘intelligent outcast’ ‘labelled because of their expertise and lack of social skills’ ‘socially awkward and overly intellectual (…) prone to obsessive interests’^11, 12, 13^). Therefore, we labelled our composite measure of non-verbal intelligence, restrictive interests and reduced need to fit in with the peer group as ‘geek index’ (GI).

We validated our measure of GI by verifying its scope to predict future academic achievement. Although the GI components were all independently associated with school results, we show they are most beneficial when co-occurring in the ‘geek’ cluster.

Finally, we tested the hypothesis that advancing paternal age is associated with higher GI in offspring. Using data from the Twin Early Development Study (TEDS), a British, population-representative study of twins, allowed us to further examine sex differences, as well as genetic and environmental contributions to this index.

## Materials and methods

### Sample

The study used the TEDS sample, a large nationally representative cohort of British twins.^14^ There were 12 468 twins from 6234 families assessed at the age of 12, with data on relevant questionnaires available for 8601 (4528 families; questionnaire details below). Out of these, 7781 (4097 families) individuals had complete data on all covariates. This subset of individuals had higher mean maternal and paternal ages than those recorded in the full sample (<1 year difference for both), lower CAST scores (6% difference), higher Raven’s Matrices test results (1% difference) and higher academic achievement at age 16 (4% difference; Supplementary Table S1). All sample descriptives are presented in Supplementary Table S1.

### Measures

GI was computed using child’s scores of (i) non-verbal intelligence, (ii) restrictive and repetitive behaviours (RRBs) and (iii) social aloofness. Scores on the Raven’s Standard Progressive Matrices^15^ test were used to obtain (i). Childhood Autism Spectrum Test^16^ (CAST) scores were used to obtain both (ii) and (iii). CAST measures came from parental reports (see Supplementary Table S2 for the list of items), and the cognitive tests were completed by the TEDS participants via web platforms; all assessments took place at the age of 12.

Covariates in the adjusted models included socioeconomic status (SES; index of parent qualifications and employment, and mother’s age at birth of first child), maternal age at conception, sex and zygosity.

Education achievement was measured using the results of nationally standardised exams taken in the United Kingdom typically at the age of 16 in a wide range of subjects (General Certificate of Secondary Education, GCSE). The curriculum and awarding of GCSEs is overseen by examination boards regulated by the British government, ensuring consistent standards across the country and throughout the time. All pupils are required to sit exams in English Language and Literature, Mathematics and Science, but are otherwise free to choose among a number of other subjects.

Science, technology, engineering and mathematics (STEM) subjects refer to the part of the school curriculum that focuses on development of the knowledge and skills essential for a number of science and technology-related careers. As such, they have been the focus of the educational policies in both United Kingdom and United States.^17, 18, 19^ High attainment in the STEM subjects have been consistently shown to predict future income over and above overall school performance, with STEM degree majors earning on average more than $15 000 per year than non-STEM degree majors in full-time jobs.^20, 21^

### Geek index

GI scores were computed by multiplying the non-verbal cognitive scores with the sum of the CAST social and RRBs items (after weighing down high RRB scores—see below). In order to avoid the confusion between GI and autism symptoms, and to ensure that our construct represents an advantageous trait, we recoded very high RRB scores (Supplementary Table S3), so that the highest GI scores were associated with midrange RRB values. In addition, all scales contributing to GI were linearly transformed, so that 1 represented a true score of 0, 2 a score of 1 and so on. This was done to avoid obtaining GI=0 for all individuals scoring 0 on at least one of the GI subscales, as a result of multiplication by 0. Finally, to ensure that our results did not arise due to unequal contribution of measures used to compute GI, or transformation of the RRB score described above, all analyses were also done using (i) standardised subscales and (ii) non-transformed RRB scores.

### Statistical analysis

All analyses were conducted using R^22^ (version 3.2.2) and OpenMX.^23^

#### Validation of the GI

To establish whether GI can predict future academic attainment, we looked at the association between GI and school results at the age of 16. The measure of academic performance reflected a product of grade score and qualification value, in approximate number of GCSE equivalents (A levels completed at this age were assumed to be double the workload of GCSEs). This enabled us to obtain an index of school attainment for all individuals regardless of the qualification taken. Given the evidence that attainment in STEM subjects is a stronger predictor of future income than attainment in Art subjects, we also investigated the association between GI and (i) number of STEM GCSEs taken, (ii) mean grades at STEM GCSEs and (iii) probability of obtaining A or A* in at least two STEM GCSEs. The effect of GI on academic performance was tested using linear and logistic mixed models fitted by maximum-likelihood procedure, with a random effect of family ID around the intercept (*lme4* package), and controlling for the effects of SES, sex, zygosity and maternal age.

We also verified whether the cognitive and behavioural traits used to derive the GI were associated with choosing more STEM GCSEs when clustering together, rather than existing independently of each other. We defined high/low levels of the GI subscales as ±1 s.d. from the mean, and looked at mean number of STEM GCSEs in ‘geeks’ (high IQ, socially aloof, high RRBs), compared to individuals with (i) high IQ, not socially aloof and low RRBs score and (ii) low IQ, socially aloof and high RRBs score. To investigate how specific this effect was, we performed similar analyses for GCSEs in Art subjects.

Finally, we used structural equation modelling (lavaan package) to verify whether a latent construct underlain by RRBs score, non-verbal IQ and social aloofness are associated with school results at age 16.

#### Association between paternal age and GI

The association between paternal age (treated as a continuous variable) and GI was investigated using mixed models, in line with the procedures described above. Both linear and quadratic models were investigated, and a log-likelihood ratio test was used to evaluate whether adding quadratic component significantly improved the fit of the model. To validate that the association between paternal age and GI is stronger than that between paternal age and the GI subscales, we compared the size and significance level of paternal age effects on the standardised GI subscales.

Given the evidence for the sexually dimorphic nature of the paternal age effects,^1, 24^ we tested whether the link between GI and paternal age differs between males and females. Furthermore, to ensure that our results did not arise due to either very limited number of offspring of fathers in the highest paternal age categories (paternal age >51) or enrichment of autism cases among the offspring of older men, all analyses were also conducted in (i) a subset of children with fathers aged 50 or younger at conception, and (ii) after excluding individuals with a confirmed autism diagnosis.

#### Mediation between paternal age and education outcomes

We tested the association between association paternal age and conception, applying mixed models methodology, as specified above. Next, we investigated whether this association is mediated by the effects of paternal age on GI. In line with the other procedures, this was done using a multilevel framework (mediation package), thus controlling for the family structure in our data. Paternal age was specified as a predictor, GI as a mediator and exam results as the outcome, with the covariates including SES, maternal age, zygosity and—where appropriate—sex. For each model, we run 100 simulation and used a bootstrap procedure to estimate 95% Quasi–Bayesian confidence intervals of the effect size estimates. Testing our hypothesis of sexually dimorphic effects of APA, we run the mediation analysis separately for male and female offspring.

#### Effects of paternal age on heritability of GI

We derived tetrachoric correlations for GI in mono- (MZ) and dizygotic (DZ) twins in OpenMx, using raw data full information maximum-likelihood estimation. Given the high MZ correlation (which was more than twice the DZ correlations and suggestive of dominance effects), we ran both ADE and AE univariate twin models to obtain the standardised genetic and environmental variance components for the trait. For more details on these models see Rijsdijk and Sham,^25^ and Neale and Maes.^26^

Subsequently, we ran a basic GxE model^27^ on GI with paternal age as moderator. This allowed us to partition the genetic and environmental effects on GI into those that are moderated and those that are independent of paternal age. As the moderating effects on the variance components in this model could be biased by a (genetic) correlation between moderator and GI, paternal age effects were regressed out of the GI scores prior to fitting the GxE model. This model allowed us to get an insight into the mechanisms behind the association between paternal age and GI—due to perfect correlation of paternal age in MZ and DZ twins, it would not be possible to formally test the genetic and environmental contributions to this association otherwise.

## Results

### GI validation

GI was approximately normally distributed in the TEDS sample (Supplementary Figure S1). Descriptive statistics are presented in Table 1. Pearson correlation coefficients indicated weak correlations between the measures underlying the GI (Supplementary Table S4).

Using results from nationally standardised exams taken in a wide range of subjects by all 16-year-old students in the United Kingdom (GCSE) allowed us to verify that GI derived from behavioural and cognitive measures collected at age 12 was predictive of overall academic achievement at age 16, controlling for the key covariates (*β*=0.17, *P*<2.26E−16). This association remained significant when GI was computed using standardised subscales (*β*=0.80, *P*<2.26E−16) and non-transformed RRB scores (*β*=0.13, *P*<2.26E−16).

GI was also positively associated with specific STEM GCSEs’ performance indicators that were previously shown to be associated with future income.^20, 28^ These included: number of STEM GCSEs (*β*=0.01, *P*=9.78E−15) mean result in each STEM exam (*β*=0.01, *P*<2.2E−16) and probability of obtaining A and A* grades for at least two STEM subjects (odds ratio=1.04 (95% confidence interval (CI): 1.03–1.04)). Overall, offspring of men >50 at conception were 32% more likely to achieve two greater than A grades compared to offspring of men aged <25 years. All analyses were adjusted for SES, sex and zygosity.

**Figure 1:**
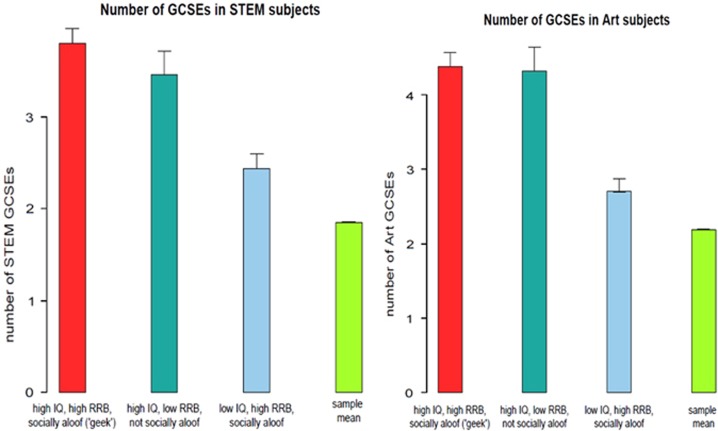
Number of STEM and Arts GCSE exams taken at age 16 in individuals with ‘geek’ cluster of traits (red bar), as well as those with high IQ or high RRB/socially aloof traits outside of the cluster (dark and light blue bars). Green bar represents the grand mean of GCSE exams taken in the whole sample. GCSE, General Certificate of Secondary Education; RRB, restrictive and repetitive behaviour; STEM, science, technology, engineering and mathematics.

Comparing mean number of GCSEs in STEM and Art subjects taken by individuals with high/low levels of cognitive and behavioural components of GI further demonstrated that the benefits of high IQ are enhanced in the presence of other ‘geeky’ traits. ‘Geeks’ took more STEM, but not Art GCSEs, compared with (i) those scoring high on the cognitive measures only, (ii) those scoring high on the behavioural measures only and (iii) sample mean (Figure 1). Structural equation model confirmed positive association between latent ‘geek’ construct and school results (*β*=0.03, *P*<0.001).

### Association between paternal age and GI

Table 1 presents GI score by parental age categories. We observed a linear trend for higher GI in the offspring of older fathers (*β*=0.13, *P*=2.26E−3), but not older mothers (*β*=0.03, *P*=0.52; Table 1), after adjusting for the family structure present in the data. The association between paternal age and GI persisted after controlling for maternal age, sex, zygosity and SES (Table 2). Paternal age effects on the full cluster of GI traits were also more significant than those on any of the standardised GI subscales, after controlling for the key covariates (Supplementary Table S5).

Sex-specific analyses showed that the paternal age effect on GI was specific to male offspring (Supplementary Table S2). These patterns persisted in the subset of offspring with fathers aged 50 or younger at conception (Supplementary Table S6) and after excluding individuals with a confirmed autism diagnosis (Supplementary Table S7), thus limiting the possibility that our results were confounded by the outliers arising due to small sample size or enrichment of autism cases in the category of the oldest fathers. A likelihood ratio test indicated that adding a quadratic component did not significantly improve the model fit (ΔlogLik=3, df=1, *P*=0.08), therefore we present the results from the linear models.

All associations remained significant when GI was computed using standardised subscales of GI (Supplementary Table S8) and non-transformed RRB scores (Supplementary Table S9).

### Paternal age, GI and education outcomes

We found a positive association between paternal age at conception and education outcomes at age 16 (*β*=0.19, *P*=4.56E−4). Multilevel mediation model revealed that this effect was fully mediated by GI in male, but not female offspring, controlling for the effects of SES, maternal age and zygosity (Table 3).

### Heritability of the GI

The much higher correlations for GI in MZ pairs (*R*_MZ_=0.59, 95% CI: 0.56–0.63) compared to DZ twins (*R*_DZ_=0.16, 95% CI: 0.12–0.20), suggested fitting a model with both additive (A) and dominant (D) genetic effects. The ADE model indicated strong dominant and relatively weak additive genetic effects (Supplementary Table S10). Given the lack of power to distinguish between these two sources of genetic effects, we decided to only consider the AE model, acknowledging that the A estimate will include the dominant genetic effects thus representing the ‘broad-sense’ heritability of GI. The AE model revealed an approximately equal proportion of genetic (*h*^2^=0.57, 95% CI: 0.50–0.64) and non-shared environmental factors (*e*^2^=0.43, 95% CI: 0.36–0.50) influencing GI variance.

### Moderating effect of paternal age on the genetic variance of GI

**Figure 2:**
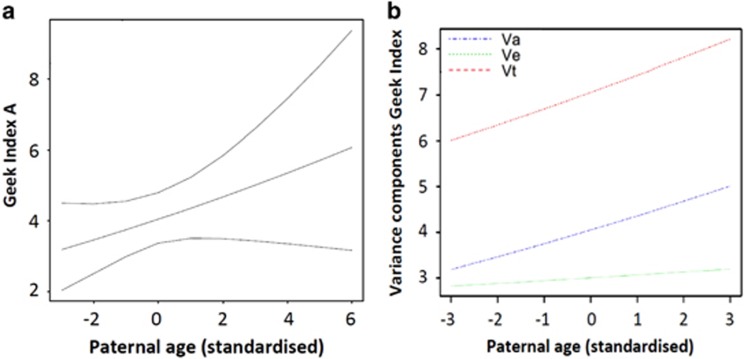
Geek index moderation by paternal age. Effects of paternal age on (**a**) genetic variance (with 95% CIs) and (**b**) total variance (Vt) split into environmental (Ve) and genetic (Va) components.

The GxE analysis suggested advancing paternal age was associated with an increase in genetic variance, effects that approached standard statistical significance (*β*=0.08, 95% CIs: −0.03 to 0.17; Figure 2) and were substantially higher than the effects of paternal age on environmental variance (*β*=0.02, 95% CIs: −0.07 to 0.11). Unstandardised A and E path estimates were, respectively, 2.01 (95% CIs: 1.84 to 2.19) and 1.73 (95% CIs: 1.61 to 1.87).

## Discussion

In summary, male offspring of older fathers had higher ‘geek index’ scores, a composite measure of high IQ, strong focus on the subject of interest and social aloofness. To our knowledge, this is the first time that APA was shown to associate with an advantageous outcome. GI was positively linked with future academic attainment—including the key predictors of future SES—suggesting a phenotypic advantage in the offspring of older fathers. Using a population-based longitudinal twin sample protects against bias and enhances generalisability, while controlling for the key covariates—including maternal age, SES and sex—and adjusting for the family structure mitigates against inflation in the reported effects.

### Molecular underpinnings of the effects of APA on GI

Our study offered new insights into the molecular mechanisms underlying the APA effects on offspring. We showed that GI was 57% heritable, highlighting the importance of the genetic factors in educational success.^29^ Both familial, additive genetic effects and *de novo* mutations—including those accumulating in paternal sperm with age—likely contributed to this measure of heritability. We acknowledge that ‘geekiness’ likely runs in families, and correlates with paternal age at conception—and therefore, the association observed in our study is underlain largely by familial factors. Nevertheless, our results suggest that genetic influences on GI may extend beyond those.

First, all associations between paternal age an GI in males remained significant after controlling for SES, which—indexing parental education and employment status—likely correlates with parental GI. Second, using a GxE model we showed that the genetic, but not environmental, contribution to GI variance increases with paternal age. In other words, genes were becoming more important in determining the GI as paternal age increased. Controlling for the genetic correlation between GI and paternal age limited the chance that the observed association arose solely due to the genetic make-up of men who decide to delay fatherhood, therefore suggesting potential contribution of *de novo* genetic factors.

Such multifactorial nature of the APA effects—with relatively minor contribution of *de novo* effects—is in line with the results from recent population genetics model,^30^ and represents a consensus between multiple lines of evidence from molecular and epidemiological research.^7, 31, 32, 33^ Although these studies focused largely on the mechanisms of association between APA and neurodevelopmental disorders, as we explain below, they can provide useful insights in unravelling the factors underlying the link between paternal age and GI.

### ‘Geekiness’, autism and talent

It is possible that the factors mediating the association between advanced paternal age and GI are overlapping with those between advanced paternal age and autism.^1, 32, 34, 35^ Although autism is a complex neurodevelopmental disorder that is qualitatively different from being a ‘geek’, it is possible that some of its facets are captured by the index measured in the current study. Indeed, original descriptions of autistic disorders by Kanner (1943) and Asperger (1944) involved a notion that the associated personality characteristics are relevant for development of high-level skills (‘astounding vocabulary of the speaking children, the excellent memory for events of several years before, the phenomenal rote memory for poems and names, and the precise recollection of complex patterns and sequences, bespeak good intelligence’ (Kanner, 1943^36^); ‘the problems are compensated by a high level of original thought and experience. This can often lead to exception achievements later in life’ (Asperger, 1944, translated by Uta Frith^37^). Several studies point towards high prevalence of individuals with the broad autism phenotype in certain professional groups, including academics, engineers and musicians.^38, 39, 40, 41, 42^ Furthermore, superior performance on non-verbal cognitive tasks in individuals on the autism spectrum versus controls has been shown before,^43^ and common genetic variants for autism were predictive of higher IQ in the general population.^44^

We showed that GI was positively associated with educational attainment, including the measures particularly predictive of future SES, and fully mediated the positive association between paternal age and school results. Although we could mitigate against the results being biased by the inclusion of individuals with a diagnosis of autism, it is likely that the effects of APA on autism symptomatology in offspring are continuously distributed in the general population,^2, 45^ contributing to the patterns reported in the current study. If advanced paternal is associated with familial and *de novo* molecular events that contribute to autism, it could also contribute to the ‘cognitive strengths’ associated with the liability to this disorder—and our results capture some of this signal. Of particular interest here is the recent evidence suggesting that familial risk for autism can be partitioned into several components, including a cluster of variants contributing to high IQ.^46^

Although our focus here remained on the beneficial effects of APA, we must emphasise they are likely to be specific to the modern knowledge-based environment, without necessarily having such advantageous effects historically. In other words, offspring of older fathers are not inherently biologically ‘fit’ rather, the cluster of behavioural and cognitive traits, which we showed they are enriched for, conveys an advantage in modern times. This is supported by a recent study investigating offspring fitness in relation to paternal age in historical and current cohorts, with a total sample size of over 1.3 million and data spanning four centuries.^47^ Although APA was strongly associated with increased infant mortality in pre-industrial populations, this relationship was reversed in the 20th-century Sweden, suggesting relative recency of the phenomenon of APA-related advantage.

### Sexual dimorphism of the effects of APA and the ‘geek index’

The notion that APA mediated geekiness and autism via partly overlapping pathways fits in with our observations of sexually dimorphic effects of APA on GI. Previous studies consistently reported higher rates of autism in males versus females,^48, 49^ with suggestions of a female protective factor contributing to this gender imbalance.^50^ Such protective effects have yet to be characterised, with mechanisms proposed to date including X chromosome-mediated effects,^51^ role of hormones,^52, 53^ differential gene expression^54^ and social factors.^55^ Nevertheless, how these mechanisms could interact with the familial and *de novo* genetic factors for autism/GI discussed above remains to be elucidated.

The notion of sexually dimorphic effects of APA on offspring has received some attention before, however, the evidence emerging from the research to date remains inconclusive. The majority of studies reporting sex differences in the APA effects suggested higher risk for APA-related disorders in female than in male offspring, with up to three-fold increase in risk in girls compared to boys.^1, 56, 57^ However, neither the meta-analysis of APA-schizophrenia studies by Miller *et al.*,^57^ nor multi-cohort autism study by Sandin *et al.*^58^ confirmed the presence of such differences.

### Comparison with previous findings

At first, our findings seem to be at odds with previous research suggesting an association between APA and lower cognitive skills. However, the results to date have been mixed, with some studies reporting lack of APA effects on IQ,^59^ and others a decrease in cognitive performance in the offspring of aged fathers.^50, 51^ In the largest study reporting effects of paternal age on educational attainment, D’Onofrio *et al.*^34^ showed that higher paternal age was associated with better school performance (D’Onofrio appendix) in population-based analyses. In our study, advanced paternal age was associated with higher scores on the Raven’s Progressive Matrices test (data not shown), therefore the lack of agreement between our findings and the literature cannot be attributed to compounding the non-verbal IQ with other measures. Discrepancies in research could be explained by different ages of the populations studied, differences in definitions of both the outcome measure and the covariates (particularly SES), and analytical methods. It is worth noting that results regarding maternal age contribution to offspring cognitive outcomes are similarly inconclusive, with literature providing the evidence for either direction of its effects.^60, 61^

### Strengths and limitations

Our novel hypothesis, analytical methods and use of a large, population-representative sample of twins, are the main strengths of our study. Controlling for SES and maternal age, we could ensure that our results did not arise solely due to higher levels of education, better financial status in men who conceive later in life or maternal factors. Furthermore, using a mixed-model approach allowed us to leverage the unique twins data set without the risk of inflating results. Finally, we verified that our findings were not driven by outliers in the sample, and remained significant after excluding individuals of very old fathers (>50 years) or those with a diagnosis of autism.

The study has, however, several limitations. It was not possible to determine whether the advantageous effects of GI extend beyond secondary education, and correlate with future SES. It remains possible that personality traits associated with GI cease to be advantageous later in life. In career settings, cognitive flexibility and ability to tune in to subtle social cues may convey more benefits than strong focus and pursuing one’s goal irrespective of others’ opinion. Although we could show that GI was strongly associated with measures of educational attainment in STEM subjects, which has been previously shown to strongly predict future income, we could not test these effects directly because cohort members are young. We also could not verify whether high GI is associated with any adverse outcomes, for example, worse socioemotional functioning, and we acknowledge that the ‘geek’ phenotype has yet to be fully characterised. Finally, we could not control for paternal age at birth of his first child. Although this would be a useful predictor of the socioeconomic index composite and would help verify the GI in offspring of men who delay versus those who extend fatherhood, relevant data had not been collected.

## Conclusions

Results of our study indicate that APA is associated with some phenotypic advantage in male offspring. A cluster of behavioural and cognitive traits identified as characteristic of ‘geeks’ was strongly predictive of academic, and thus likely also career success. We could show that paternal age is more strongly associated with such combination of traits than any of these traits alone, suggesting a special relationship between APA and educational attainment. Such an association is likely to be mediated by factors similar to those linking APA and autism. Our results provide original insights into the possibility of phenotypic advantage associated with delayed fatherhood.

## Figures and Tables

**Table 1:** Descriptive statistics for GI

	*Mean (s.e.)*	N
	40.5 (0.2)	8361
*Sex*		
Females	38.8 (0.3)	4591
Males	42.5 (0.3)	3779
		
*Zygosity*		
MZ	38.7 (0.3)	3043
DZ	41.5 (0.3)	5299
		
*Paternal age (years)*		
<25	39.6 (1.1)	303
25–34	40.0 (0.3)	4443
35–44	41.1 (0.4)	2620
45–50	43.2 (1.5)	246
>50	46.6 (2.5)	57
		
*Maternal age (years)*		
<20	38.5 (2.4)	67
21–30	40.3 (0.3)	3597
31–40	40.5 (0.3)	4272
>40	40.2 (1.2)	239
		
*Autism*		
Affected	40.1 (3.8)	36
Non-affected	40.5 (0.2)	7633

Abbreviations: DZ, dizygotic; GI, geek index; MZ, monozygotic.

**Table 2:** Crude and adjusted association between paternal age and geek index in the entire sample, and males and females separately

*Offspring sex*	*Crude*			*Adjusted*		
	β *(s.e.)*	P	n	β *(s.e.)*	P	n
M+F	0.13 (0.04)	2.27E−3	7669	0.16 (0.05)	3.28E−3	7434
M	0.17 (0.07)	9.98E−3	3469	0.28 (0.09)	1.02E−3	3361
F	0.11 (0.06)	4.27E−2	4200	0.09 (0.07)	2.08E−1	4073

Table presents the effect size (*β*) with its standard error and significance value, and size of the sample used in the analyses. The covariates in the adjusted model included maternal age, socioeconomic status, sex and zygosity. All analyses were adjusted for the family structure present in the data.

**Table 3:** Results of the multilevel mediation analysis on the effects, between paternal age and educational achievement via geek index, controlling for SES, sex (in M+F analysis), zygosity and maternal age

	*Causal mediation effects*		*Direct effects*		*Total effects*	
	*Estimate (95% CI)*	P	*Estimate (95% CI)*	P	*Estimate (95% CI)*	P
M+F	0.03 (0.01 to 0.05)	<0.001	0.03 (−0.13 to 0.18)	0.52	0.06 (−0.10 to 0.21)	0.34
M	0.04 (0.01 to 0.06)	<0.001	0.08 (−0.14 to 0.33)	0.58	0.12 (−0.10 to 0.35)	0.38
F	0.02 (−0.01 to 0.04)	0.22	−0.03 (−0.21 to 0.16)	0.82	−0.01 (−0.20 to 0.17)	0.98

Abbreviation: SES, socioeconomic status.

Quasi–Bayesian 95% CIs and *P*-values are presented together with estimates of the effect sizes of the respective associations.
