# Supplementary material for: Advantageous developmental outcomes of advancing paternal age
Source: Transl Psychiatry. 2017 Jun 20;7(6):e1156–. doi: 10.1038/tp.2017.125 (PMC5537646; doi:10.1038/tp.2017.125)
Supplement: Supplementary Material [file tp2017125x1.pdf]

SUPPLEMENTARY MATERIAL

	Full sample	Subsample
age	11.31 (0.01)	11.21 (0.01)
% females	50	55
paternal age	32.80 (0.04)	33.37 (0.06)
maternal age	30.23 (0.03)	31.14 (0.05)
CAST score	5.01 (0.03)	4.69 (0.04)
Raven's matrices score	11.07 (0.03)	11.21 (0.04)
GCSE results (/equivalent)	98.23 (0.23)	102.45 (0.31)
A-level results (/equivalent)	321.90 (1.31)	336.24 (1.86)
N	12,468	8,601

Table S1. Comparison between the full TEDS sample tested at age 12 and the subset of individuals included in the adjusted analyses (no missing data on the key variables). Means of the variables are presented with their standard errors for both the full and subsample.

DOMAIN	ITEM
social	Is it important to him/her to fit in with the peer group?<br>Are people important to him/her?<br>Does s/he care how s/he is perceived by the rest of the group?
RRB	Does s/he appear to notice unusual details that others miss?<br>Does s/he like to do things over and over again, in the same way all the time?<br>Does s/he mostly have the same interests as his/ her peers? (reversed)<br>Does s/he have an interest which takes up so much time that s/he does little else?<br>Does s/he appear to have an unusual memory for details?<br>Does s/he have any unusual and repetitive movements?<br>Does s/he try to impose routines on him/herself, or on others, in such a way that it causes problems?

Table S2. CAST items included in the "geek index" computation.

RRB score	
actual	adjusted
0	1
1	2
2	3
3	4
4	3
5	2
6	1
7	0.8

Table S3. Adjustment of CAST RRBs scores for computing the GI.

	Restrictive repetitive behaviors	Social aloofness	Raven's Progressive Matrices
**Restrictive repetitive behaviors**	-	-	-
**Social aloofness**	(a) r=-0.17, p<2.2E-16<br>(b) r=0.17, p<2.2E-16	-	-
**Raven's Progressive Matrices**	(a) r=-0.06, p=1.82E-08<br>(b) r=-0.06, p=9.23E-07	(a) r=-0.04, p=7.61E-05<br>(b) r=-0.04, p=1.81E-4	-

Table S4. Pearson product correlation coefficients between measures used to compute GI, with their p-values. (a) represent values obtained from the full sample, and (b) from the individuals with paternal age data.

	ß (SE)	p
Geek index	0.008 (0.003)	3.28E-3
Social aloofness	0.002 (0.003)	0.459
Restrictive, repetitive behaviours	0.008 (0.003)	9.92E-3
Non-verbal intelligence	0.005 (0.003)	0.088

Table S5. Effects sizes and significance levels for the association between paternal age and standardized measures of geek index and its subscales, controlling for the effects of SES, sex, zygosity and maternal age.

	crude			adjusted		
	ß (SE)	p	n	ß (SE)	p	n
M+F	0.11 (0.02)	$1.81E^{-2}$	7612	0.14 (0.06)	$1.78E^{-2}$	7381
M	0.12 (0.07)	$7.52E^{-2}$	3440	0.22 (0.09)	$1.28E^{-2}$	3334
F	0.09 (0.06)	$1.12E^{-1}$	4172	0.07 (0.07)	$3.07E^{-1}$	4047

Table S6. Crude and adjusted association between paternal age and geek index in the males and females combined and separately, offspring with fathers aged >= 50 at conception. Table presents the effect size (ß) with its standard error and significance value, and size of the sample used in the analyses. The covariates in the adjusted model included maternal age, socioeconomic status, sex and zygosity.

	crude			adjusted		
	ß (SE)	p	n	ß (SE)	p	n
M+F	0.13 (0.04)	3.25E-03	7633	0.16 (0.06)	4.15E-03	7399
M	0.15 (0.02)	2.28E-02	3440	0.26 (0.09)	2.57E-03	3333
F	0.12 (0.06)	3.51E-02	4193	0.10 (0.16)	1.6E-01	4066

Table S7. Crude and adjusted association between paternal age and geek index in the males and females combined and separately, excluding individuals with a confirmed diagnosis of autism. Table presents the effect size (ß) with its standard error and significance value, and size of the sample used in the analyses. The covariates in the adjusted model included maternal age, socioeconomic status, sex and zygosity.

	crude			adjusted		
	ß (SE)	p	n	ß (SE)	p	n
M+F	0.03 (0.01)	1.46E-03	7612	0.04 (0.01)	3.41E-03	7381
M	0.04 (0.02)	5.67E-03	3440	0.06 (0.02)	8.19E-04	3334
F	0.02 (0.01)	7.90E-02	4172	0.02 (0.02)	2.75E-01	4047

Table S8. Crude and adjusted association between paternal age and geek index in the males and females combined and separately, using standardized measures to compute GI. Table presents the effect size (ß) with its standard error and significance value, and size of the sample used in the analyses. The covariates in the adjusted model included maternal age, socioeconomic status, sex and zygosity.

	crude			adjusted		
	ß (SE)	p	n	ß (SE)	p	n
M+F	0.17 (0.05)	5.53E-04	7612	0.20 (0.06)	1.31E-03	7381
M	0.25 (0.08)	1.02E-03	3440	0.40 (0.10)	3.63E-05	3334
F	0.11 (0.06)	7.63E-02	4172	0.05 (0.08)	4.76E-01	4047

Table S9. Crude and adjusted association between paternal age and geek index in the males and females combined and separately, using non-transformed RRB measures to compute GI. Table presents the effect size (ß) with its standard error and significance value, and size of the sample used in the analyses. The covariates in the adjusted model included maternal age, socioeconomic status, sex and zygosity.

	estimate	CIs
h^2	0.03	0-0.18
d^2	0.56	0.40-0.62
e^2	0.41	0.38-0.44

Table S10. Standardized ADE components of the Geek Index.

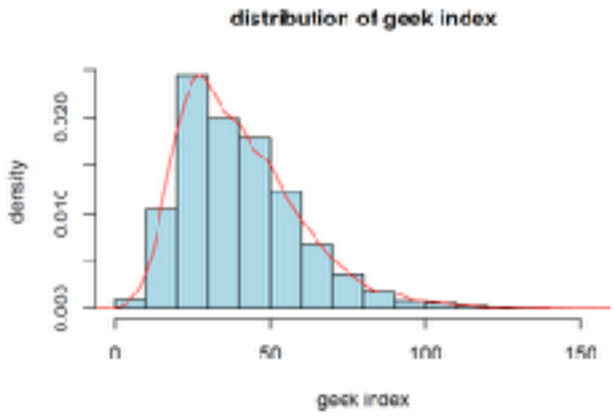

Figure S1. Distribution of the geek index in the TEDS sample.
